# Limited variability in upper thermal tolerance among pure and hybrid populations of a cold-water fish

**DOI:** 10.1093/conphys/cow063

**Published:** 2016-12-15

**Authors:** Zachery R. R. Wells, Laura H. McDonnell, Lauren J. Chapman, Dylan J. Fraser

**Affiliations:** 1 Department of Biology, Concordia University, Montreal, Quebec, Canada H4B 1R6; 2 Department of Biology, McGill University, Montreal, Quebec, Canada H3A 1B1

**Keywords:** Climate change, critical thermal maximum, hybridization, population size, thermal tolerance

## Abstract

As climate warming threatens the persistence of many species and populations, it is important to forecast their responses to warming thermal regimes. Climate warming often traps populations in smaller habitat fragments, not only changing biotic parameters, but potentially decreasing adaptive potential by decreasing genetic variability. We examined the ability of six genetically distinct and different-sized populations of a cold-water fish (brook trout, *Salvelinus fontinalis*) to tolerate acute thermal warming and whether this tolerance could be altered by hybridizing populations. Critical thermal maximum (CT_max_) assays were conducted on juveniles from each population to assess thermal tolerance, and the agitation temperature was recorded for assessing behavioural changes to elevated temperatures. An additional metric, which we have called the ‘CT_max_–agitation window’ (CT_max_ minus agitation temperature), was also assessed. The CT_max_ differed between five out of 15 population pairs, although the maximal CT_max_ difference was only 0.68°C (29.11–29.79°C). Hybridization between one large population and two small populations yielded no obvious heterosis in mean CT_max_, and no differences in agitation temperature or CT_max_–agitation window were detected among pure populations or hybrids. Summer variation in temperature within each stream was negatively correlated with mean CT_max_ and mean CT_max_–agitation window, although the maximal difference was small. Despite being one of the most phenotypically divergent and plastic north temperate freshwater fishes, our results suggest that limited variability exists in CT_max_ among populations of brook trout, regardless of their population size, standing genetic variation and differing natural thermal regimes (temperature variation, minimum and maximum). This study highlights the level to which thermal tolerance is conserved between isolated populations of a vertebrate species, in the face of climate warming.

## Abstract (precis)

To forecast the impact of climate warming on cold-water fishes, thermal tolerance trials were conducted on six populations of brook trout from Cape Race, Newfoundland, Canada. Of these, three were outcrossed to assess the effect of hybridization. Although differences were found, there was little variation among populations in how they responded to thermal stress.

## Introduction

Human-induced climate change may be the single greatest threat to global biodiversity (Sala *et al*., 2000). Climate change can interact with habitat fragmentation by creating physical (i.e. drought) or physiological (i.e. temperature) barriers (Hughes, 2000; Walther *et al*., 2002; Pearson and Dawson, 2003; Travis 2003), which limit the potential for independently mobile organisms to shift habitats. As fragments become smaller, so too do the populations within them, resulting in a loss of genetic diversity via an increased likelihood of inbreeding, genetic drift and reduced gene flow (Young *et al*., 1996; Keller and Largiader, 2003; Andersen *et al*., 2004; Ezard and Travis, 2006). In general, this process reduces adaptive potential, the ability of a population to tolerate environmental change, and further decreases population size. This study was designed to investigate the ability of isolated and different-sized populations to deal with climate warming, and the extent to which hybridization might enhance this response.

By increasing genetic variability within a population upon which natural selection can act, hybridization may improve population responses to climate warming, such as upper thermal tolerance (Stockwell *et al*., 2003; Pickup *et al*., 2012). Nevertheless, a number of factors influence the outcomes of hybridization, which may also decrease or have no effect on average fitness within populations (Edmands, 2007; Fraser *et al*., 2008). In part, such outcomes depend on population size; larger populations are expected to provide more genetic material than small ones, and small populations are therefore expected to benefit greatly from hybridization due to having lower genetic variability and possibly reduced phenotypic plasticity prior to hybridization (Lande, 1988; Ellstrand and Elam, 1993; Frankham, 1996; Reed and Frankham, 2003; but see Wood and Fraser, 2015; Wood *et al*., 2015). Therefore, when studying population responses to climate warming, it is important to consider both the relative benefits of population size and hybridization concurrently.

A practical metric for assessing the thermal tolerance of individuals from different, fragmented populations of a species is the critical thermal maximum (CT_max_; but see Elliott, 1981), defined as the temperature at which an organism can no longer maintain coordinated movement or equilibrium control (Becker and Genoway, 1979). In nature, a loss of equilibrium affects an organism's ability to forage or avoid predation, which may ultimately affect individual fitness. As acclimation temperature (*T*_a_) has been found to be correlated positively with CT_max_ (Cox *et al*., 1974; Zhang and Kieffer, 2014; McDonnell and Chapman, 2015, although see Galbreath *et al*., 2004; Recsetar *et al*., 2012), the ancestral history and origin of a population are thought to be linked to an organism's ability to tolerate temperature increases (Stockwell *et al*., 2003; McDermid *et al*., 2012) and, as such, stream temperature regimes may result in population-specific thermal tolerance. A relatively new metric to assess thermal tolerance, agitation temperature, described by McDonnell and Chapman (2015) as the temperature at which a fish first begins to exhibit refugia-seeking behaviour (circling of the chamber, seeking refuge in substrate), may also provide insight to how quickly individuals can sense and attempt to react to environmental change. In addition, the difference between these two traits (CT_max_–agitation window) may represent a fitness metric yet unexplored in thermal tolerance literature.

Salmonids are a socioeconomically important family of cold-water fishes having traditional and commercial value. Although they are rich in populations and occupy a diverse range of habitats, habitat fragmentation has depleted their numbers, and their viability is of growing concern as climate change warms northern regions (Walther *et al*., 2002; Alley *et al*., 2003; Hinzman *et al*., 2005). Recently, studies on the ability of salmonids to tolerate climate warming have been variable, with some finding evidence of population-level variation in thermal physiology (Eliason *et al*., 2011) and others finding little or none (Elliott and Klemetsen, 2002; Kelly *et al*., 2014). In particular, the brook trout (*Salvelinus fontinalis*) is an extremely diverse (Angers *et al*., 1995; Wood *et al*., 2015) and highly plastic (Hutchings, 1996; Imre *et al*., 2002) stenotherm, inhabiting a thermal window of 1–22°C (Xu *et al*., 2010) to maintain both an internal body temperature below 20°C (Scott and Crossman, 1973) and physiological pathways affecting individual growth, reproductive timing, foraging and predator avoidance (De Staso and Rahel, 1994; Magoulick and Wilzbach, 1998). With such a low thermal window, cold water species such as brook trout might be strongly affected by climate change, as global temperature is expected to increase by 0.7–7.4°C over the course of the 21st century (Rouse *et al*., 1997; Heino *et al*., 2009). Additionally, northern brook trout populations may be at an adaptive disadvantage due to being genetically depauperate as a result of isolation in glacial refugia and historical bottlenecks (Bernatchez and Wilson, 1998). Few studies have investigated the thermal performance of brook trout, with many among them having looked only at: (i) the effects of ploidy, heating rate, or interspecific differences with other salmonids; (ii) thermal tolerance at a static upper thermal limit; or (iii) comparing few populations with long histories of hatchery manipulation (McCauley, 1958; Benfey *et al*., 1997; Galbreath *et al*., 2004; McDermid *et al*., 2012; Stitt *et al*., 2014). More research is needed in order to prepare for, and adequately address, the effects of climate change on this socioeconomically important species at the intraspecific scale, taking into account population size and hybridization.

**Figure 1:**
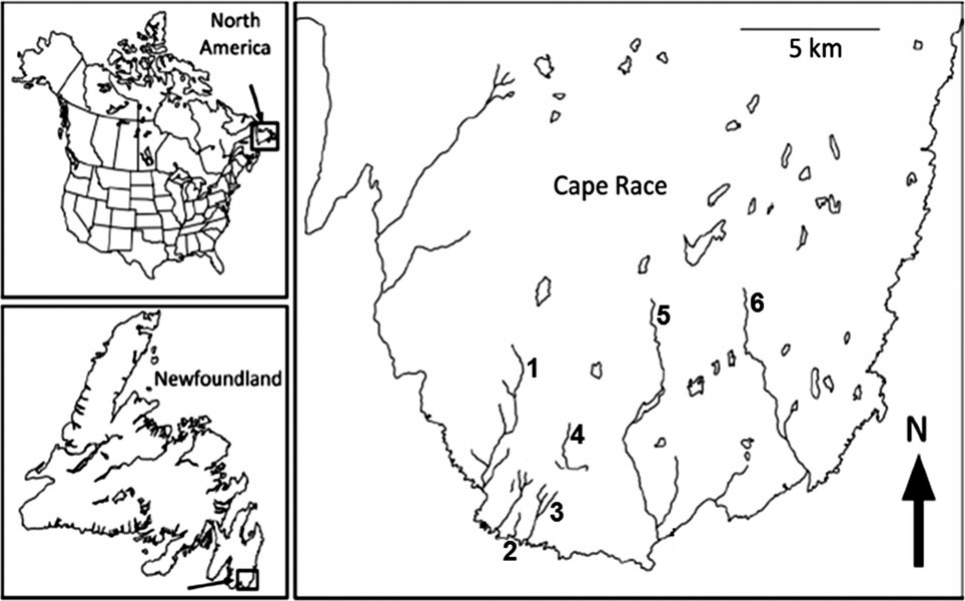
The geographical locations of study streams in Cape Race, Newfoundland, Canada: (1) Freshwater (FW); (2) Still There By Chance (STBC); (3) Whale Cove (WC); (4) Ouananiche Beck (OB); (5) Watern Cove (WN); and (6) Cripple Cove (CC).

Our study used six fragmented, genetically distinct populations of brook trout occupying streams on Cape Race (CR), Newfoundland, Canada (Fig. 1), to explore effects of hybridization and population size on upper thermal tolerance. Fragmentation of CR streams occurred as a result of the late-Wisconsinan glaciation (10–12 000 years before present; Danzmann *et al*., 1998), and these populations have been studied extensively (Hutchings, 1991; Fraser *et al*., 2014; Wood and Fraser, 2015; Wood *et al*., 2015). Besides having a common ancestry, CR trout populations have a number of additional attributes for such a study. First, the small size of CR streams (ranging in length from 0.27 to 8.10 km) allows for thorough sampling and accurate estimates of population size. Second, CR populations range greatly in size, with *N* = 780–5120 and *N*_*b*_ = 27–200 (where *N* is the census population size and *N*_*b*_ the effective number of individuals breeding in one spawning season; an analogue of effective population size that is positively related to genetic diversity of a population; Bernos and Fraser, 2016). Third, consistent with theory, small CR populations have less neutral genetic variation than large CR populations (Fraser *et al*., 2014), yet genetic variation underlying quantitative traits does not vary with population size (Wood *et al*., 2015); such apparent discrepancies make this population system an intriguing one for investigating what genetic metrics best predict responses to environmental change. Finally, CR streams vary in their thermal regimes (Supplementary material Fig. S1), with those inhabited by the two smallest populations in the present study having the coldest overall mean monthly temperatures (Table 1).

**Table 1:** Monthly mean annual temperatures (in degrees Celsius) and standard deviations of six streams in Cape Race, Newfoundland across years 2012–15, ordered by increasing genetic population size (*N*_*b*_; harmonic mean), with mean *N*_*b*_ and adult census population size (*N*; harmonic mean) based on data from 2012–15 (range of annual point estimates in parentheses; Bernos and Fraser, 2016)

	STBC	WC	OB	CC	WN	FW
January	4.02 (1.59)	3.47 (1.98)	1.14 (1.26)	0.91 (0.71)	4.33 (1.83)	3.19 (2.14)
February	3.66 (1.49)	3.54 (1.92)	1.18 (1.22)	0.91 (0.71)	4.38 (1.74)	2.86 (1.91)
March	3.55 (1.29)	3.42 (1.71)	1.44 (1.25)	1.04 (0.92)	4.4 (1.59)	2.71 (1.74)
April	4.07 (1.30)	3.76 (1.7)	3.04 (2.12)	3.16 (2.34)	5.11 (1.43)	3.3 (1.57)
May	5.48 (1.61)	6.39 (2.23)	7.47 (2.44)	7.71 (2.63)	6.2 (0.80)	5.31 (2.06)
June	7.56 (2.73)	8.84 (3.41)	10.43 (3.45)	10.26 (3.3)	6.59 (1.88)	8.94 (3.79)
July	9.18 (2.68)	12.40 (2.58)	15.57 (3.10)	13.63 (3.1)	14.01 (2.64)	14.04 (3.32)
August	9.94 (2.44)	13.58 (2.37)	16.69 (2.60)	15.78 (2.94)	14.76 (2.38)	15.49 (2.75)
September	9.44 (2.27)	12.25 (2.48)	14.52 (2.59)	14.18 (3.17)	12.84 (2.34)	13.69 (2.84)
October	7.67 (1.13)	8.89 (1.99)	10.15 (2.5)	12.12 (4.99)	9.28 (1.80)	9.80 (2.15)
November	6.51 (0.96)	6.63 (1.37)	5.86 (2.22)	5.94 (2.44)	6.72 (0.64)	7.26 (1.47)
December	4.78 (1.87)	4.74 (2.09)	3.19 (1.93)	2.22 (2.00)	5.42 (1.20)	5.04 (2.09)
*N*_*b*_	27.65 (14–66)	31.36 (23–52)	62.26 (41–95)	73.54 (65–99)	178.59 (110–267)	200.05 (173–237)
*N*	916.87 (587–1405)	783.09 (530–1148)	2568.76 (1940–3835)	1862.08 (1471–2412)	2836.00 (1003–8416)	5118.30 (4024–6514)

Abbreviations: CC, Cripple Cove; FW, Freshwater; OB, Ouananiche Beck; STBC, Still There By Chance; WC, Whale Cove; and WN, Watern Cove.

The upper thermal tolerance of pure and hybrid individuals were compared in terms of CT_max_, agitation temperature and a new metric coined the ‘CT_max_–agitation window’, i.e. the difference between an individual's CT_max_ and agitation temperature. A smaller window signifies that an individual can continue to carry out normal behaviours for a longer period before demonstrating avoidance behaviour in increasing temperatures, whereas a large window indicates that the individual displays avoidance behaviour earlier and, as such, regular behaviours are disrupted sooner. We hypothesized that large populations would have higher thermal tolerance (i.e. higher CT_max_, higher agitation temperature and smaller CT_max_–agitation window) due to more genetic variation, as greater genetic variation may increase the likelihood of more thermally tolerant individuals, and small populations would have limited thermal tolerance as a result of both lower genetic diversity and colder thermal regimes in the wild (Table 1 and Supplementary material Fig. S1). We also hypothesized that the magnitude of population size difference would affect that relationship; specifically, hybridizing small populations with a large one would disproportionally benefit the small populations, as they might have reduced fitness because of inbreeding depression.

## Materials and methods

### Procuration of brook trout

From 13 to 26 October 2014, gametes were collected from six CR populations: Cripple Cove (CC), Freshwater (FW), Ouananiche Beck (OB), Still There By Chance (STBC), Whale Cove (WC) and Watern Cove (WN). For larger streams, individuals were collected from previously documented spawning sites (four to six per stream) and from areas observed to have obvious redd formations and large brook trout aggregates (Wood *et al*., 2014). For smaller streams, wherein fish densities were lower in the spawning grounds, individuals were collected throughout the entire stream.

Potential spawning individuals were collected via electrofishing surveys and checked for ‘readiness’; a release of sperm for males, and an elongated cloaca/soft belly for females. Readiness was assessed in the days leading up to the expected date of gamete collection, and ready fish were held for 24–72 h in flow-through cages before collection.

Gamete collection took place between 19.00 and 01.00 h. Sperm was collected in 1.5 ml microcentrifuge tubes, whereas eggs were collected in 60 ml opaque plastic containers. Gametes were kept on ice and insulated so as not to freeze, and transported to St John's, Newfoundland, immediately after collection. They were then flown directly to Montreal, and crossed within 15 h from the beginning of gamete collection. Crosses were conducted to produce pure population offspring as well as full-reciprocal F1 hybrids (Supplementary material Table S1). Families were incubated separately within mesh-bottom containers 5.2 cm in diameter placed randomly with respect to population within a single 1000 litre recirculating tank and maintained at 7.0 ± 0.3°C (mean ± SD). Eggs were left mostly undisturbed until the eyed stage, except to remove fungal eggs, to reduce potential mortality following fertilization, at which point dead individuals were counted and removed daily. Dissolved oxygen and pH did not differ in different tank locations and were consistently maintained throughout the experiment.

After reaching yolk absorption, separate brook trout families were kept in flow-through bins within two larger, identical, 3000 litre tanks prior to thermal tolerance trials. The water temperature was maintained between 15.5 and 16.5°C (±0.2°C, SD), and multiple air stones ensured dissolved oxygen saturation. pH was 7.5 (±0.2, SD) across all tanks, and artificial light was set at a natural daylight cycle (corresponding to St John's, Newfoundland). Tanks were cleaned daily, fish were fed *ad libitum* two times daily, feeding time was constant, and all fish were kept in the same thermal conditions from fertilization to the end of the thermal tolerance experiments. At the time of the experiment, fish were ~2–4 months post-yolk absorption.

### Upper thermal tolerance trials

Subjecting an organism to a linear increase in temperature, the onset of spasms and loss of equilibrium are used as markers for CT_max_, with loss of equilibrium being the most commonly used (Lutterschmidt and Hutchison, 1997). Since its introduction (Cowles and Bogert, 1944), CT_max_ studies have evolved to account for a number of factors influencing CT_max_ results. A low rate of temperature increase (e.g. 0.02°C/min) allows organisms to acclimate to rising temperatures, whereas a high rate of temperature increase (e.g. 1°C/min) results in core body temperature lag, skewing CT_max_ results (Cox *et al*., 1974; Becker and Genoway, 1979; Galbreath *et al*., 2004).

Experimental fish were starved for 24 h prior to trials, and trials were performed at the same time daily to ensure similar metabolic rates (Clark *et al*., 2013). Fish were given 1 h of acclimation time after being moved to a rectangular experimental test tank (60 cm × 32 cm × 30 cm; length × width × height) to reduce stress associated with handling and to acclimate to minute changes in water temperature. Significant efforts were made to standardize starting water temperature, which ranged from 16.20 to 17.97°C over 34 trials. Each trial consisted of two pure trout from the same family and two maternal hybrids, with a total of *n* = 122 trout tested across 61 families from six populations (Table 2). Within the larger rectangular test tank, four smaller, tapered circular flow-through chambers (14 cm top diameter × 10 cm bottom diameter × 11.5 cm deep) were used to hold each experimental fish. Rock substrate was provided in each chamber to act as potential fish refuge. During the trial, individuals were subjected to a constant (0.3°C/min) increase in water temperature that was controlled, monitored and recorded by a temperature-control unit and software (TMP-REG, AutoResp; Loligo Systems; McDonnell and Chapman, 2015). Agitation temperatures were recorded for each fish as the point where an obvious shift in behaviour first occurred. For brook trout in this study, fish generally remained relatively still as temperatures increased until a point (agitation temperature), after which they began to circle the chamber hurriedly or sought refuge in substrate, or a combination of both behaviours. This agitation temperature, along with temperature at CT_max_, were both confirmed after each experiment using time-stamped video footage taken via a mounted webcam. Immediately after loss of equilibrium, fish were removed and placed in an aerated recovery chamber until regaining equilibrium and normal opercular movement; total length (in millimetres) was then recorded.

**Table 2:** Number of families used per population (pures) and per cross-type (hybrids) in the experiment

Pure or hybrid	Maternal population	Paternal population	Number of families	Number of individuals
Pure	CC	CC	4	8
Pure	FW	FW	7	14
Pure	OB	OB	9	18
Pure	STBC	STBC	4	8
Pure	WC	WC	8	16
Pure	WN	WN	10	20
Hybrid	FW	STBC	2	4
Hybrid	STBC	FW	4	8
Hybrid	FW	WC	5	10
Hybrid	WC	FW	8	16

Two unique individuals were used from each family across the six populations, and each reciprocal hybrid cross-type.Abbreviations: CC, Cripple Cove; FW, Freshwater; OB, Ouananiche Beck; STBC, Still There By Chance; WC, Whale Cove; and WN, Watern Cove.

### Statistical analysis

All analyses were conducted using R (R Core Team, 2016), and all packages were retrieved from its open-source directory. Linear mixed models were used to determine whether CT_max_, agitation temperature or CT_max_–agitation window differed across populations and between pure fish and their corresponding hybrids. Two models were run for each hybrid comparison and for the pure data alone. Model type ‘A’ initially included length and cross-type as independent fixed effects, whereas model ‘B’ included length, population size and the percentage coefficient of variation for summer temperature (summer CV) in each stream and for all years of data available. Hybrid comparison ‘B’ models did not include population size due to smaller data sets. All models were run using mean values of each of the three thermal tolerance traits, as well as using the family-level (within-population) mean variance for each trait. Factor significance was determined by reverse model selection using the R package pbkrtest to compare complex models with less complex ones using *F*-tests (Halekoh and Højsgaard, 2014). Mother identity was included as a random effect to account for maternal effects on thermal tolerance and to incorporate the replication of trials (two trials per family). For each model, data were normally distributed (variances logged), and pairwise *P*-values were calculated and corrected for false discovery rates (Benjamini and Hochberg, 1995) using the R package lsmeans (Lenth, 2015).

## Results

**Figure 2:**
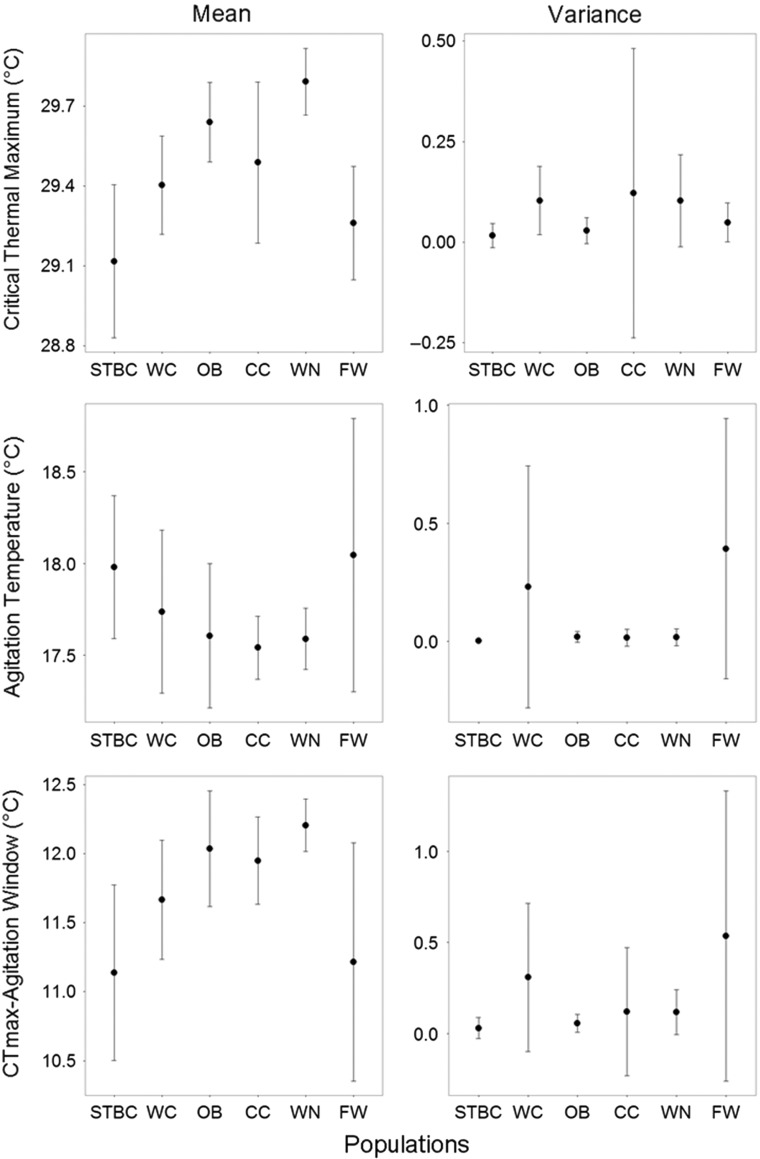
Mean values and mean variance of critical thermal maximum (CT_max_), agitation temperature and CT_max_–agitation window with 95% confidence intervals for six brook trout populations from Cape Race, Newfoundland, Canada, in order of ascending effective number of individuals breeding in one spawning season (*N*_*b*_). The maximal difference in mean CT_max_ is 0.68°C, agitation temperature is 0.51°C, and CT_max_–agitation window is 1.79°C. Abbreviations: CC, Cripple Cove; FW, Freshwater; OB, Ouananiche Beck; STBC, Still There By Chance; WC, Whale Cove; and WN, Watern Cove.

**Figure 3:**
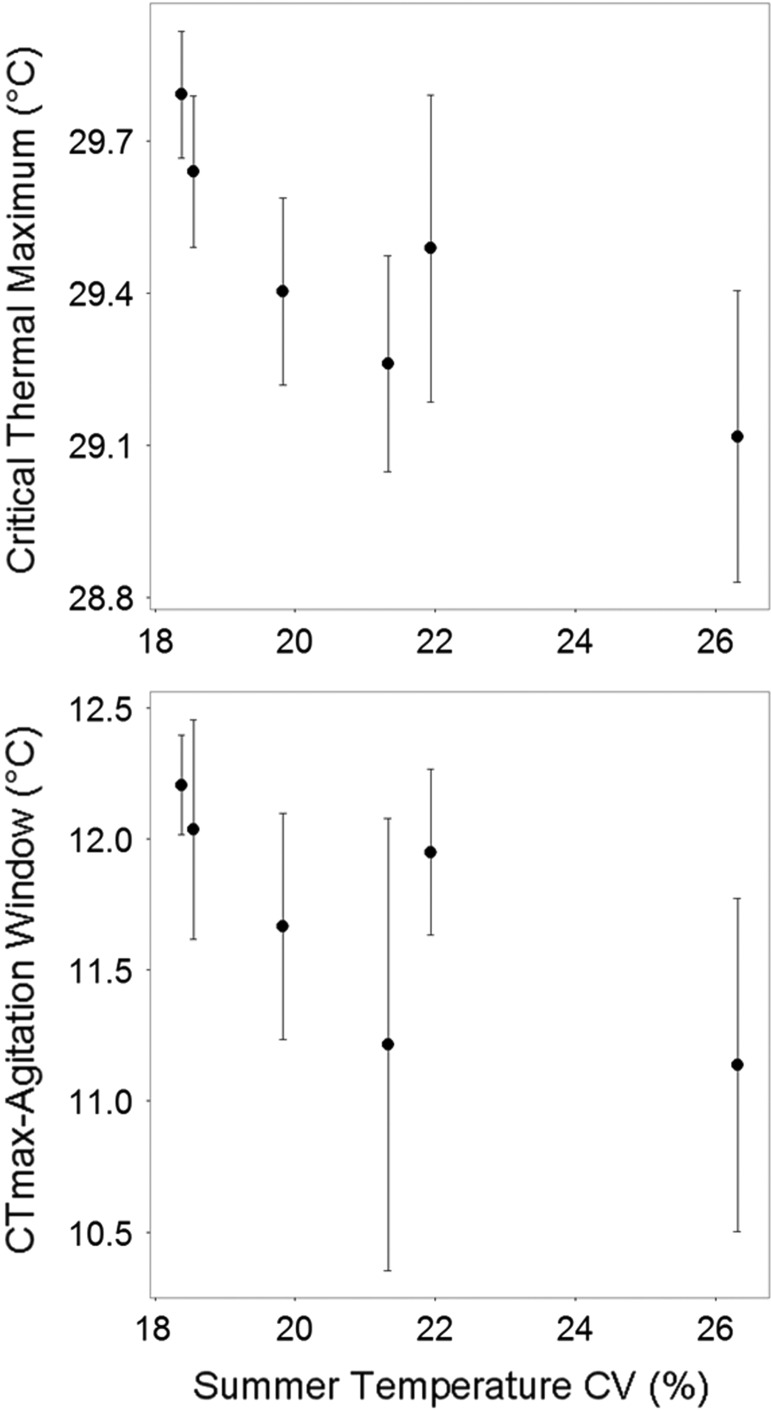
Mean critical thermal maximum (CT_max_) and mean CT_max_–agitation window plotted with 95% confidence intervals by the percentage coefficient of variation (CV) in summer temperature (from July to September) for all years of data available.

Mean CT_max_ was significantly different between five out of fifteen pure population comparisons (overall effect: d.f. = 32.96, *F* = 5.44, *P* < 0.001). Plots of 95% confidence intervals for mean CT_max_ by population (Fig. 2) showed a maximal difference in mean CT_max_ of 0.68°C; WN had significantly higher mean CT_max_ than STBC (d.f. = 34.11, *F* = 5.44, *P* < 0.001), WC (d.f. = 33.62, *F* = 5.44, *P* = 0.02) and FW (d.f. = 33.72, *F* = 5.44, *P* < 0.001) and OB had significantly higher CT_max_ than STBC (d.f. = 35.04, *F* = 5.44, *P* = 0.01) and FW (d.f. = 35.04, *F* = 5.44, *P* = 0.03; Table 3). Mean agitation temperature and CT_max_–agitation window did not differ between all other pure populations, and the variance of all traits did not differ between pure populations (Fig. 2). Summer CV (thermal variation) had a significant effect on only mean CT_max_ (d.f. = 38.42, *F* = 18.10, *P* < 0.001) and mean CT_max_–agitation window (d.f. = 38.13, *F* = 5.60, *P* = 0.02), with larger variance resulting in lower thermal tolerance (i.e. lower CT_max_ and larger CT_max_–agitation window; Fig. 3). Length and population size had no effect on mean and variance values of thermal tolerance for pure populations (Table 3A) and were therefore not included in the final models.

**Table 3:** Summary of linear mixed model results for three thermal tolerance traits between pure populations (**A**) and pure vs. hybrid comparisons (**B**)

Measured trait	Model	Factor	*F*-statistic	Degrees of freedom	Pairwise comparison	*P*-value
(A)						
CT_max_	Pure A	Length	0.04 (0.63)	75.18 (40.00)		0.84 (0.43)
		Cross-type	5.44 (1.04)	32.96 (33.93)		**0.00** (0.26)
				29.61 (18.15)	CC–FW	0.37 (0.99)
				29.23 (16.83)	CC–OB	0.37 (0.99)
				30.58 (21.82)	CC–STBC	0.17 (0.99)
				29.40 (17.42)	CC–WC	0.77 (0.97)
				28.52 (14.21)	CC–WN	0.17 (0.97)
				35.04 (35.65)	FW–OB	**0.03** (0.97)
				35.04 (35.65)	FW–STBC	0.43 (0.99)
				35.04 (35.65)	FW–WC	0.37 (0.97)
				33.72 (32.64)	FW–WN	**0.00** (0.97)
				35.04 (35.65)	OB–STBC	**0.01** (0.99)
				35.04 (35.65)	OB–WC	0.17 (0.97)
				33.54 (31.99)	OB–WN	0.37 (0.97)
				35.04 (35.65)	STBC–WC	0.17 (0.97)
				34.11 (33.82)	STBC–WN	**0.00** (0.97)
				33.62 (32.30)	WC–WN	**0.02** (0.99)
	Pure B	Length	2.53 (0.63)	62.78 (40.00)		0.12 (0.43)
		*N*_*b*_	0.09 (0.15)	36.67 (36.94)		0.77 (0.70)
		Summer CV	18.10 (0.52)	38.42 (34.91)		**0.00** (0.48)
Agitation temperature	Pure A	Length	0.00 (3.53)	63.10 (24.22)		0.99 (0.07)
		Cross-type	0.46 (2.10)	33.84 (34.15)		0.80 (0.09)
	Pure B	Length	0.07 (3.53)	79.41 (24.22)		0.79 (0.70)
		*N*_*b*_	0.33 (0.90)	37.15 (36.29)		0.57 (0.35)
		Summer CV	1.13 (3.38)	38.11 (38.86)		0.29 (0.07)
CT_max_–agitation window	Pure A	Length	0.02 (3.76)	65.77 (1.20)		0.90 (0.27)
		Cross-Type	1.69 (1.23)	33.81 (33.97)		0.16 (0.32)
	Pure B	Length	0.57 (1.47)	80.9 (1.52)		0.45 (0.38)
		*N*_*b*_	0.16 (0.00)	36.56 (36.78)		0.69 (0.98)
		Summer CV	5.60 (0.31)	38.13 (38.81)		**0.02** (0.58)
(B)						
CT_max_	Hybrid 1A	Length	3.01 (8.13)	24.47 (5.41)		0.10 (**0.03**)
		Cross-type	0.46 (4.30)	19.55 (4.84)		0.72 (0.08)
				23.99 (3.78)	FW–FWSTBC	0.86 (0.08)
				21.02 (3.67)	STBC–STBCFW	0.41 (0.05)
	Hybrid 1B	Length	3.01 (8.13)	24.47 (5.41)		0.10 (**0.03**)
		Summer CV	0.01 (0.99)	9.30 (9.24)		0.92 (0.35)
	Hybrid 2A	Length	6.46 (0.04)	54.00 (21.15)		**0.01** (.85)
		Cross-type	1.36 (1.89)	32.54 (16.40)		0.27 (0.17)
				41.12 (15.70)	FW–FWWC	0.35 (0.62)
				39.31 (10.96)	WC–WCFW	0.75 (0.19)
	Hybrid 2B	Length	6.46 (0.45)	54.00 (22.51)		**0.01** (0.51)
		Summer CV	2.87 (0.77)	12.58 (13.67)		0.11 (0.39)
Agitation temperature	Hybrid 1A	Length	0.69 (0.411)	25.24 (11.52)		0.41 (0.53)
		Cross-type	3.37 (2.07)	19.89 (8.07)		**0.04** (0.18)
				22.15 (9.84)	FW–FWSTBC	**0.02** (0.76)
				21.00 (4.95)	STBC–STBCFW	0.93 (0.49)
	Hybrid 1B	Length	1.79 (2.06)	23.91 (6.48)		0.19 (0.20)
		Summer CV	0.02 (5.48)	9.23 (7.40)		0.88 (0.05)
	Hybrid 2A	Length	2.64 (0.28)	50.56 (22.57)		0.11 (0.60)
		Cross-type	0.51 (0.98)	32.73 (15.92)		0.68 (0.43)
				40.54 (14.73)	FW–FWWC	0.68 (0.85)
				39.01 (10.45)	WC–WCFW	0.68 (0.31)
	Hybrid 2B	Length	2.64 (0.44)	50.56 (4.68)		0.11 (0.51)
		Summer CV	0.35 (2.62)	12.80 (13.94)		0.56 (0.13)
CT_max_–agitation window	Hybrid 1A	Length	2.96 (1.26)	23.74 (8.27)		0.10 (0.29)
		Cross-type	1.04 (1.62)	19.82 (8.21)		0.40 (0.26)
				22.33 (9.78)	FW–FWSTBC	0.14 (1.00)
				21.00 (4.93)	STBC–STBCFW	0.67 (0.48)
	Hybrid 1B	Length	2.96 (2.96)	23.74 (6.77)		0.10 (0.13)
		Summer CV	0.02 (2.32)	9.21 (7.40)		0.89 (0.17)
	Hybrid 2A	Length	0.01 (0.17)	47.45 (22.35)		0.93 (0.68)
		Cross-type	1.14 (3.20)	33.36 (16.11)		0.35 (0.05)
				40.60 (15.16)	FW–FWWC	0.41 (0.14)
				39.01 (10.60)	WC–WCFW	0.73 (0.12)
	Hybrid 2B	Length	0.00 (0.15)	50.17 (22.51)		0.95 (0.70)
		Summer CV	1.06 (0.71)	13.04 (13.67)		0.32 (0.41)

A total of 18 linear mixed models were conducted for mean values of each trait, and an additional 18 for the within-population family-level variance for each trait (in parentheses). Model variant ‘A’ assessed the effects of length and cross-type, whereas variant ‘B’ assessed effects of length and summer coefficient of variation (CV) for hybrid comparisons, and additionally population size (*N*_*b*_) for pures. Values in bold are significant at *P* < 0.05. Abbreviations: CC, Cripple Cove; CT_max_, critical thermal maximum; FW, Freshwater; OB, Ouananiche Beck; STBC, Still There By Chance; WC, Whale Cove; and WN, Watern Cove.

**Figure 4:**
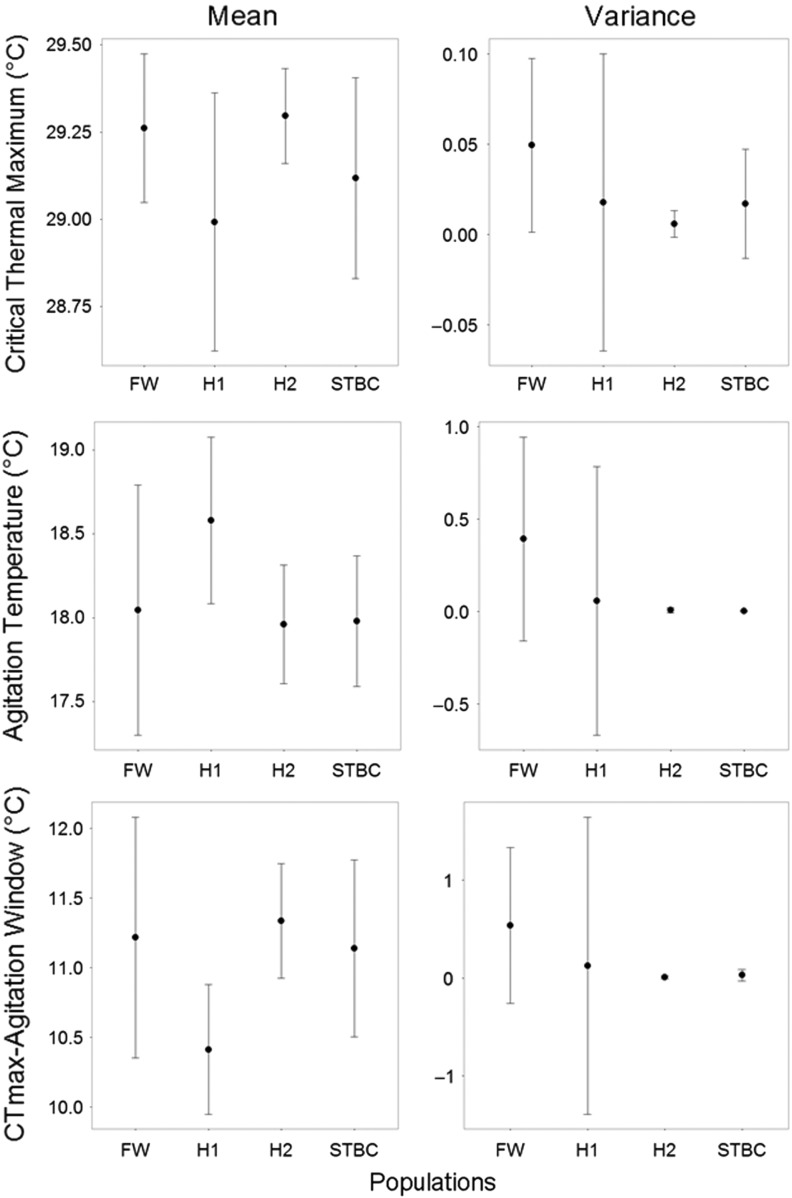
Means and 95% confidence intervals for critical thermal maximum (CT_max_), agitation temperature and CT_max_–agitation window (mean response values and mean variance) for one pure–hybrid comparison. Freshwater (FW; large population) was crossed with Still There By Chance (STBC; small population). A statistically significant difference in mean CT_max_ was found between FW and H1 (d.f. = 22.15, *F* = 3.37, *P* = 0.02). H1 and H2 represent reciprocal F1 hybrid crosses.

Length had a significant effect on thermal performance (Table 3B) in some models for pure vs. hybrid comparisons; however, this effect was not specific to one trait, nor the trait's measure (i.e. mean or variance). Of all traits, only one comparison of thermal tolerance (mean CT_max_, FW vs. FW-STBC hybrid) was found to show a weak significant difference (d.f. = 22.15, *F* = 3.37, *P* = 0.02). However, mean CT_max_ differed by a maximum of 0.3°C between different hybrid cross-types (Fig. 4, Supplementary material Fig. S2 and Table 3B). No effect of summer CV was found for any pure vs. hybrid comparisons.

Supplementary visual analyses were conducted to determine whether any relationship existed between the dependent variables CT_max_, agitation temperature and CT_max_–agitation window and the year-long variation in temperature, mean maximal and minimal summer temperatures in each stream across all years for which data were collected. Of these, none was found to have an obvious relationship with any of the dependent variables (Supplementary material Fig. S3).

## Discussion

The aim of this study was to investigate how varyingly-sized and genetically distinct populations of a cold-water species respond to acute thermal warming, and how hybrids created with these populations respond in comparison. Our study on brook trout found significant differences in mean CT_max_ between some pure populations and significant effects of summer CV on mean CT_max_ and mean CT_max_–agitation window. Interestingly, populations that had greater variability in summer temperatures exhibited lower fitness, i.e. lower CT_max_ and larger CT_max_–agitation windows. However, no population differences in agitation temperature or CT_max_–agitation window were seen (means or variances), no heterosis was observed, nor was there any effect of population size on any of the traits (means or variances). Although some studies have found similar evidence for intraspecific variation in upper thermal tolerance in salmonids (Eliason *et al*., 2011; Gradil, 2015), including brook trout (McDermid *et al*., 2012; Stitt *et al*., 2014), others have found no differences between populations (Elliott and Klemetsen, 2002), differences between hybrid and pure crosses (Fields *et al*., 1987), evidence for heterosis in thermal tolerance of copepods (Willett, 2012) and evidence for increased survival of heterozygotes at near-lethal temperatures in Eastern mosquitofish (*Gambusia holbrooki*; Meffe *et al*., 1995). To our knowledge, agitation temperature has been assessed in fishes only once before (McDonnell and Chapman, 2015) but was studied in relationship to sex and acclimation temperature.

Our study examined six genetically distinct populations of brook trout that have been isolated, without gene flow or human disturbances, for potentially 12 000 years (Danzmann *et al*., 1998). Previous studies have found that although these CR brook trout populations differ nearly 50-fold in census size *N* and 10-fold in effective number of breeders *N*_*b*_ (Bernos and Fraser, 2016), there is no evidence for differences in (i) quantitative genetic variation and trait differentiation in relationship to population size, nor (ii) phenotypic plasticity in relationship to population size (Wood and Fraser, 2015; Wood *et al*., 2015). Therefore, our study provides further evidence that population size may not be tightly related to the ability of a population to respond to environmental change, and that thermal tolerance, in particular (physiologically and behaviourally), may be highly conserved even in such a plastic species as *S. fontinalis*. A key factor supporting this is that some of our populations fall below what many deem a minimal viable population (MVP) size for long-term persistence, which is hypothesized to range from *N* = 4100 to 7300 (Table 1; Reed *et al*., 2003; Traill *et al*., 2007). It might then be expected that populations exceeding this size would show greater thermal tolerance, but we found little supporting evidence for this. Thus, while the second largest population (WN: mean *N*_*b*_ = 178.59 and mean *N* = 2836.00) had the highest CT_max_ and one of the two smallest populations had the lowest (STBC: mean *N*_*b*_ = 27.65 and mean *N* = 916.87), the largest population (FW: mean *N*_*b*_ = 200.05 and mean *N* = 5118.30) also had the second lowest CT_max_. Another expected outcome might be that hybridizing between populations above and below the minimal viable population threshold would benefit smaller populations disproportionally. In our hybridized crosses, one large population (FW, see above) was hybridized with two smaller populations (STBC, see above; WC: mean *N*_*b*_ = 31.36 and mean *N* = 783.09; Bernos and Fraser, 2016) with little effect on any thermal tolerance trait, whether measured by mean or variance.

Owing to the considerable number of populations we compared, hybridizing between populations vastly different in population size and annual thermal regimes (Supplementary material Fig. S1) and assessing both mean values and variance in each trait, our results suggest that thermal tolerance (in terms of temperature tolerance and behavioural responses to temperature increases) seems to be highly conserved in *S. fontinalis* and remains relatively unchanged across isolated populations and in their hybrid offspring. Even the significant mean differences found between our populations support this theory, as their magnitude is likely not to be biologically meaningful (0.68°C; see Fields *et al*., 1987 for a similar discussion), nor their variances smaller in smaller populations. Allowing our populations to acclimate at a temperature regularly experienced during summer months in the wild (16°C; Table 1 and Supplementary material Fig. S1), measuring CT_max_ in a fluctuating thermal environment (Ketola and Saarinen, 2015), increasing temperature at a rate previously found to be optimal for fish studies on thermal tolerance (0.3°C/min; Becker and Genoway, 1979) and measuring both CT_max_ and a behavioural metric of agitation temperature have provided new evidence for less variability in thermal tolerance than previously thought. Reasons for this may be the scale at which other studies were conducted (see McDermid *et al*., 2012; Stitt *et al*., 2014) as well as the historical genetic or environmentally driven similarities in the populations being assessed. In our case, although populations experience different thermal regimes (Table 1 and Supplementary material Fig. S1) and have been isolated for thousands of years, the relative geographical proximity of the populations may have resulted in similar environmental pressures shaping their ability to cope with climate warming. Nonetheless, we would expect smaller populations to have reduced variance in thermal tolerance and therefore reduced adaptive potential; however, this was not the case. It is possible that at larger scales, between-population and pure vs. hybrid differences in thermal tolerance may have been larger. Additionally, similar thermal performance may have to do with the highly conserved natures of heat shock proteins (for example, see Molina *et al*., 2000; Basu *et al*., 2002; Gradil, 2015) as well as similar haematocrit and peak heart rate values across populations (Gradil, 2015). As these proteins have increased expression in thermal crises, their naturally high level of conservation may be correlated with a highly conserved CT_max_. A next research step could be to acclimate these trout populations to different temperatures and then measure CT_max_, to determine whether acclimation potential is reduced with population size.

Our study is one of only a few to have examined upper thermal tolerance in a large number of populations of cold-water fishes (see also Eliason *et al*., 2011; McDermid *et al*., 2012; Stitt *et al*., 2014) and is the first to assess the CT_max_–agitation window. Additionally, we have accounted for genetic population size and family-level variation and tested for the effects of population mixing, which are factors that may affect the degree of tolerance. We found little population differentiation in upper thermal tolerance and no indication that population size or hybridization (enhancing genetic variability) affects thermal tolerance. It is, however, difficult to disentangle the effects of temperature regime from population size, as the two show a weak positive correlation. We have therefore highlighted the potential for a highly plastic and divergent species to have lower than expected resilience in the face of climate warming; large or mixed populations are not necessarily conferred any greater resilience to climate warming than small, isolated populations, nor do they provide increased resilience to small populations via hybridization. Although we cannot completely disentangle the relative roles of historical genetic vs. environmentally driven similarities in the populations being assessed, our results are a cause for concern for the general conservation of this and related cold-water species as the climate warms.
